# Objective measurement of sedentary time and physical activity in people with rheumatoid arthritis: protocol for an accelerometer and activPAL^TM^ validation study

**DOI:** 10.31138/mjr.30.2.125

**Published:** 2019-06-29

**Authors:** Ciara M. O’Brien, Joan L. Duda, George D. Kitas, Jet J. C. S. Veldhuijzen van Zanten, George S. Metsios, Sally A. M. Fenton

**Affiliations:** 1 School of Sport, Exercise and Rehabilitation Sciences, University of Birmingham, Birmingham, United Kingdom,; 2 Department of Rheumatology, Russells Hall Hospital, Dudley Group NHS Foundation Trust, West Midlands, United Kingdom,; 3 Faculty of Education, Health and Wellbeing, University of Wolverhampton, Wolverhampton, United Kingdom

**Keywords:** Validation, ActiGraph, accelerometer, activPAL, rheumatoid arthritis, sedentary behaviour, physical activity

## Abstract

**Background:**

The accurate measurement of sedentary time and physical activity in Rheumatoid Arthritis (RA) is critical to identify important health consequences and determinants of these behaviours in this patient group. However, objective methods have not been well-validated for measurement of sedentary time and physical activity in RA.

**Aims:**

Specific objectives are to: 1) validate the ActiGraph GT3X+ accelerometer and activPAL3^μTM^ against indirect calorimetry and direct observation respectively, and define RA-specific accelerometer cut-points, for measurement of sedentary time and physical activity in RA; 2) validate the RA-specific sedentary time accelerometer cut-points against the activPAL3^μTM^; 3) compare sedentary time and physical activity estimates in RA, using RA-specific vs. widely-used non-RA accelerometer cut-points.

**Methods:**

*Objective 1:* People with RA will wear an ActiGraph GT3X+, activPAL3^μTM^, heart rate monitor and indirect calorimeter, whilst being video-recorded undertaking 11 activities representative of sedentary behaviour, and light and moderate intensity physical activity. *Objectives 2 and 3:* People with RA will wear an ActiGraph GT3X+ and activPAL3^μTM^ for 7 days to measure free-living sedentary time and physical activity.

**Discussion:**

This will be the first study to define RA-specific accelerometer cut-points, and represents the first validation of the ActiGraph accelerometer and activPAL^TM^, for measurement of sedentary time and physical activity in RA. Findings will inform future RA studies employing these devices, ensuring more valid assessment of sedentary time and physical activity in this patient group.

## INTRODUCTION

There exists a wealth of research documenting levels of physical activity participation in diverse populations, and reporting the health benefits of engagement in light (1.6–2.9 metabolic equivalents [METs]) and moderate-to-vigorous (≥3 METs) intensity physical activity for specific groups.^1–9^ More recently, research has begun to examine the levels of engagement in sedentary behaviour (waking behaviour expending ≤1.5 METs whilst sitting/reclining/lying),^10,11^ in order to understand implications for health. Indeed, there is evidence to suggest that sedentary behaviour is an independent risk factor for heightened inflammation,^12,13^ incident diabetes, and all-cause, cardiovascular disease and cancer mortality^14^ in adults.

For people living with Rheumatoid Arthritis (RA), the positive effects of physical activity for pertinent RA outcomes are well-established. For example, evidence suggests that physical activity is beneficially linked to disease activity, systemic inflammation, physical function, pain, fatigue, rheumatoid cachexia outcomes, psychological wellbeing and markers of cardiovascular disease.^8,15–31^ Furthermore, new evidence suggests that sedentary behaviour may be adversely linked to disease activity, physical function and cardiovascular risk^32^ in this patient group. However, available data indicate that people with RA typically do not engage in sufficient levels of physical activity to yield positive health outcomes, and spend long periods of the day sedentary.^32,33^

Until recently, our understanding of the levels and health consequences of sedentary behaviour and physical activity in RA has largely been based on studies employing self-report methods to quantify engagement in these behaviours. The selection of self-report instruments introduces issues around measurement validity and reliability, such as social desirability bias and errors in participant recall,^32,34–36^ limiting the accuracy of such measures in sedentary behaviour and physical activity research. However, objective devices, such as accelerometers and posture sensors, are now more readily employed to quantify levels of free-living sedentary behaviour and physical activity in the general population.^34,37–40^ As such, there now exists significant opportunity to employ such instruments to the surveillance of sedentary time and physical activity in the RA population.^32^ That is, to understand dose-response relationships between sedentary time and physical activity with RA outcomes, identify salient determinants of such behaviours to be targeted in interventions, and subsequently evaluate the efficacy of such interventions for improving RA outcomes.

### Accelerometers

Accelerometers are typically small and lightweight devices, usually worn on the hip or wrist, that afford the ability to continuously monitor free-living sedentary time and physical activity.^34,39,41^ The ActiGraph accelerometer (ActiGraph, LLC., Pensacola, Florida, USA) is the most frequently employed accelerometer in field-based research.^42,43^ This device can capture human movement (accelerations) on the vertical (Y), horizontal right-left (X) and horizontal front-back (Z) axes, and these data can be used to determine the vector magnitude (VM) of these accelerations (VM = √(axisY^2^ + axisX^2^ + axisZ^2^)). Accelerations are recorded over user-defined time intervals (epochs), which are converted by the manufacturer’s software (Actilife) into ‘activity counts’. Researcher-developed algorithms (referred to as ‘cut-points’) are then applied to the accelerometer activity counts, in order to quantify time spent in different intensities of activity (sedentary behaviour, and light, moderate and vigorous intensity physical activity).

The most common accelerometer cut-point employed to assess sedentary time is ≤99 counts per minute [cpm]).^44^ This is a uniaxial (single axis) cut-point, which originates from a validation study of the ActiGraph accelerometer, conducted among adolescent girls.^45^ Following publication, the ≤99 cpm cut-point was subsequently employed in the National Health and Nutrition Examination Survey (NHANES) to estimate population prevalence of sedentary time among American adults.^46^ In conjunction, uniaxial accelerometer cut-points were employed to the NHANES data to estimate frequency and duration of light, moderate and vigorous intensity physical activity (light intensity physical activity, 100–2019 cpm; moderate intensity physical activity, 2020–5998 cpm; vigorous intensity physical activity, ≥5999 cpm) among this cohort. These physical activity cut-points were defined by Troiano et al.,^47^ on the basis of weighted averages of criteria from 4 calibration studies,^48–51^ and have since been frequently employed in studies of sedentary behaviour and physical activity in RA.^2,52^

However, more recently, researchers have started to move away from the assumption that ‘one size fits all’, and there has been an increase in the number of population-specific accelerometer cut-points developed.^53–55^ Still, researchers employing accelerometry in RA studies are heavily reliant on algorithms developed in validation studies of ‘healthy adults’,^47^ since no RA-specific accelerometer cut-points have been derived. This is particularly problematic when we consider that the physiology and associated activity patterns of people living with RA are likely to differ substantially to those among ‘healthy adults’ in the general population (eg, a relatively higher basal metabolic rate is characteristic of RA).^56^ As such, there is an urgent requirement for validation studies to develop RA-specific accelerometer cut-points to permit more accurate measurement of accelerometer-assessed sedentary time and physical activity in RA. Further, to ensure progress in this field, it is essential that the validity of these accelerometer cut-points for the measurement of free-living behaviour is established.

Despite several advantages relative to self-report, accelerometers are still limited in their ability to measure posture – an important facet of the characterisation of sedentary behaviour. That is, the established definition of sedentary behaviour stipulates a consideration of both low energy expenditure (≤1.5 METs) *and* a sitting/reclining/lying posture.^10,11^ Indeed, whilst cut-points can be applied to accelerometer data to provide an (indirect) measure of energy expenditure, accelerometers are less able to detect the posture at which low-energy behaviours are undertaken.^57,58^ In this way, the activPAL^TM^ posture sensor (PAL Technologies Ltd., Glasgow, UK) offers an advance over accelerometers for free-living assessment of sedentary time, and is currently considered the ‘gold standard’ to measure sedentary time in field-based research.^37,58–63^

### ActivPAL^TM^ posture sensor

The activPAL^TM^ is a small, lightweight device, worn attached to the front of the right thigh, in a mid-anterior position. The activPAL^TM^ has increasingly been used to measure free-living sedentary time, due to its ability to distinguish between sitting/lying and standing postures.^37,58–63^ Certainly, the activPAL^TM^ has demonstrated high validity for the measurement of sedentary time in different populations, when compared against the criterion of direct observation.^58,61,62,64,65^ Less frequently, the activPAL^TM^ is used to measure time spent stepping as an estimate of physical activity. However, the activPAL^TM^ is limited to the extent at which these data can be accurately interpreted to determine physical activity intensity, which is currently estimated based on step cadence.^66,67^ To date, only 1 study has validated the activPAL^TM^ against direct observation in the RA population.^68^ In this study, participants wore an activPAL^TM^ whilst lying, sitting, standing, walking on a treadmill, and undertaking 10 activities of daily living (ADLs [eg, reading a newspaper, washing and drying dishes, placing bed linens on pillows and duvet]). In analysis, *t-*tests indicated overall estimates of time spent sedentary, standing and stepping (seconds [mean ± standard deviation]) from the activPAL^TM^ vs. direct observation did not significantly differ. Linear regression also demonstrated a strong relationship between time spent sedentary (r = .74), standing (r = .86) and stepping (r = .93) derived from the activPAL^TM^ vs. direct observation. However, Bland and Altman^69^ explained that regressions indicating the strength of a relationship, does not provide scope to determine the degree of agreement between 2 methods. Indeed, it would be surprising to find non-significant comparability of 2 methods that measure the same variables.^69^

### Study aims

To address these critical knowledge gaps, this study will validate the ActiGraph GT3X+ accelerometer and activPAL3^μTM^ posture sensor for the measurement of free-living sedentary time and physical activity in the RA population. Specific objectives are as follows:

Objective 1: Laboratory-based validation
  - Validate the ActiGraph GT3X+ and activPAL3^μTM^ against criterion standards (indirect calorimetry and direct observation, respectively), for the measurement of sedentary time and physical activity in RA. Using the criterion of indirect calorimetry, calibrate the ActiGraph GT3X+ to define RA-specific accelerometer cut-points for sedentary time, and light and moderate intensity physical activity.
Objective 2: Field-based validation of RA-specific sedentary time accelerometer cut-points against the activPAL3^μTM^
  - Establish the validity of the new RA-specific sedentary time accelerometer cut-points for free-living assessment of sedentary time in RA. Estimates of sedentary time computed using RA-specific accelerometer cut-points, will be compared against the criterion of activPAL3^μTM^-assessed sedentary time (minutes/day).
Objective 3: Accelerometer cut-point comparison (RA-specific vs. non-RA accelerometer cut-points)
  - To compare estimates of time spent sedentary, and engaged in light and moderate intensity physical activity – specifically, to compare:
    1. Sedentary time estimates derived from widely-used ‘healthy adult’ (non-RA) accelerometer cut-points, against the criterion of activPAL3^μTM^-assessed sedentary time (minutes/day) in people living with RA.
    2. Estimates of free-living sedentary time, and light and moderate intensity physical activity (minutes/day) in people living with RA, derived using: a) the new RA-specific accelerometer cut-points vs. b) widely-used ‘healthy adult’ (non-RA) accelerometer cutpoints.^46,47^

## METHODOLOGY

### Participants and recruitment

This study has been approved by the local National Health Service Research Ethics Committee (West Midlands – Black Country Research Ethics Committee 16/WM/0371).

People with RA will be recruited from Rheumatology outpatient clinics at a hospital in Dudley, England. Eligibility criteria for this study will be: a clinical diagnosis of RA according to the American College of Rheumatology-European League Against Rheumatism Classification Criteria,^70^ aged ≥18 years old and the ability to ambulate independently without (Objective 1)/with (Objectives 2 and 3) the use of an assistive device.^68^ All participants will give informed consent, prior to initiating data collection.

### Protocol

#### Objective 1: Laboratory-based validation

Participants (target n = 20)^68^ will be asked to report to a temperature-controlled laboratory (22°C) in a fasted state (12 hours prior), having refrained from exercise for 48 hours before data collection. One hour prior to participant arrival, the indirect calorimeter (Cortex Metalyzer® 3B [Cortex Biophysik, Leipzig, Germany]) will be calibrated using Cortex Metalyzer® 3B software (MetaSoft®), in accordance with the manufacturer’s instructions (criterion standard for ActiGraph GT3X+). A video camera will be set up on a tripod overlooking the laboratory for direct observation of behaviour (criterion standard for activPAL3^μTM^).

Upon arrival, participants will undertake physical assessments, including height (cm), weight (kg), body composition (body-mass index [BMI], body fat [%], fat-free mass [kg]) and Disease Activity Score-28 (DAS-28 [Erythrocyte Sedimentation Rate plus 28 swollen-and-tender joint count]). Participants will then be fitted with the ActiGraph GT3X+, activPAL3^μTM^, Polar heart rate monitor (Polar Electro Oy Ltd., Kempele, Finland) and Cortex Metalyzer® 3B (via face mask) for the duration of the laboratory study (approximately 2 hours).

Whilst wearing this equipment, each participant will be instructed to carry out a total of 11 activities (*Table 1*), comprising a standardised testing component of 6 activities and 5 ADLs. These activities have been selected to represent various energy expenditures (METs), ranging from sedentary behaviour to light and moderate intensity physical activity.^71^ The selected ADLs have been used in previous studies aiming to replicate a free-living environment in a laboratory setting, in order to validate ActiGraph accelerometers and the activPAL^TM^ in different populations.^54,64,67,68,72,73^ Reclining, sitting and standing (standardised testing component 1) will be completed prior to the ADLs. Participants will then perform the ADLs in a random order to avoid ordering effects^54,68,74,75^ (Microsoft Excel [Microsoft Corporation, Redmond, USA] will be used to randomly sort ADLs, prior to participant arrival), and will be permitted to use their upper limbs during sit-stand transitions.^68^ Furthermore, participants will be given general, non-specific advice about how to carry out each activity, to ensure that their movement patterns during the ADLs are representative of a free-living environment.^68^ Treadmill walking (standardised testing component 2) will be completed after the ADLs.

**Table 1.** Activities undertaken during the laboratory-based validation study (Objective 1).

Standardised testing component 1	Energy expenditure (METs)

Reclining *Reclining on a hospital bed*	1.3
Sitting *Sitting on a still chair with uncrossed legs*	1.3
Standing *Standing on the floor with feet flat and arms by side*	1.3

Activities of daily living	Energy expenditure (METs)

Reading a newspaper *Sitting on a chair without a desk/table*	1.3
Washing and drying dishes *Standing whilst washing up and drying up bowls, small plates and large plates*	1.8
Ironing and folding clothes *Standing whilst taking clothes out of a laundry bag, and ironing them using an iron and mini ironing* board *Folding the ironed clothes*	2.0
Placing bed linens on pillows and duvet *Standing whilst placing a bed sheet on a single hospital bed, pillow cases on 2 pillows and a duvet cover on a single duvet*	2.5
Sweeping the floor *Using a broom to sweep up a pile of debris into a cardboard box, emptying the cardboard box and continuing cycle*	3.3

Standardised testing component 2	Energy expenditure (METs)

Walking at 3.2 km/h *On a treadmill, no incline*	2.8
Walking at 4 km/h *On a treadmill, no incline*	3.0
Walking at 4.8 km/h *On a treadmill, no incline*	3.5

MET, metabolic equivalent of task; km/h, kilometres per hour

All MET values are based on the Compendium of Physical Activities^71^

Each activity will be undertaken repeatedly for 6 minutes.^54,75^ Resting heart rate (beats per minute), VO_2_ (ml·min·kg) and METs will be measured during the 6-minute period of sitting (standardised testing component 1), and used to establish a baseline for each participant. Five-minute rest periods will be implemented to separate each of the ADLs, in order to allow heart rate and VO_2_ (ml·min·kg) to return to resting levels.^54,76^ Consecutive 1-minute rest periods will be added if these values do not return to resting levels after 5 minutes.

All equipment will be synced to ensure recording at the same time of day (MetaSoft®, video camera, Actilife and activPAL3^μTM^ software [PAL Connect]). The start and finish time of the protocol, individual activities and rest periods, will be recorded by the researcher using the time displayed on the computer interface (MetaSoft®). These times will be used to ensure accurate comparison between time-stamped raw data collected via the ActiGraph GT3X+ and activPAL3^μTM^, with criterions (VO_2_ [ml·min·kg] and METs [indirect calorimetry], and direct observation [video camera recordings]).

#### Objective 2: Field-based validation of RA-specific accelerometer cut-points

The protocol for Objective 2 of this study has been described elsewhere.^77^ Briefly, participants (target n = 100)^2,23,52^ will undertake physical measures (height [cm], weight [kg], BMI, body fat [%], fat-free mass [kg] and DAS-28). Following which, they will be asked to wear the ActiGraph GT3X+ and activPAL3^μTM^ for 7 days, for assessment of free-living sedentary time and physical activity.

#### Objective 3: Accelerometer cut-point comparison

The same protocols employed in Objective 2 will be employed to achieve Objective 3 of this study.

### Measures

#### Indirect calorimetry

The Cortex Metalyzer® 3B uses a breath-by-breath system to directly measure an individual’s concentration of inspired oxygen (O_2_) and expired carbon dioxide (CO_2_). These data are transferred to MetaSoft® in real-time, and the individual’s VO_2_ (ml·min·kg) and METs are calculated and displayed in real-time on the computer interface.

Participant details, such as biological sex, date of birth, height (cm), weight (kg) and the size of the face mask will be entered into MetaSoft®. After answering any questions, the researcher will fit the participant with the Polar heart rate monitor and face mask; the face mask will be attached to a head net and a mouthpiece turbine. The gas sensor will be fitted to the mouthpiece turbine once the participant confirms they are comfortable in the face mask.

Once the participant assumes a lying position (standardised testing component 1), they will rest for 5 minutes. Following this, once heart rate has reached steady state for 1 minute, the researcher will start data collection with MetaSoft®. The participant will be instructed to refrain from speaking at this point, but to give agreed hand signals (e.g., thumbs up/down) for the duration of data collection. At the end of the testing period (after all activities have been completed), the researcher will stop the MetaSoft® recording, and data will be exported to Microsoft Excel for further analysis.

#### Direct observation

Direct observation is commonly used when validating devices such as the activPAL3^μTM^ in different populations.^58,61,64,65,68^ In this study, participants will be video-recorded (at standard recording speed, 25 frames/second) throughout the laboratory testing procedure, in order to observe their time spent sedentary, standing, stepping (seconds), as well as number of steps and sit-stand transitions.

The video camera will start recording when the participant is lying on the bed, ready to begin the first activity. The recording will be stopped when the participant has finished the last activity.

#### ActiGraph accelerometer and activPAL3^μTM^ posture sensor

The ActiGraph GT3X+ is a triaxial accelerometer (19g; 4.6cm × 3.3cm × 1.5cm) that records accelerations on 3 axes (Y, X, Z), which are used to compute VM (VM = √(axisY^2^ + axisX^2^ + axisZ^2^)). The device can be configured to record accelerations at a sample rate of 30–100 Hertz (Hz). During data reduction, raw accelerations stored in the ActiGraph GT3X+ are processed through a digital filter using Actilife, which limits the range of frequency to 0.25–2.5 Hz. Each sample is then summed over user-defined epochs (range 1–60 seconds), which are converted (by Actilife) to activity counts. In this study, the ActiGraph GT3X+ will be configured to record accelerations in 1-second epochs, at a rate of 30 Hz. The ActiGraph GT3X+ will be vertically positioned on the right hip of each participant, attached to an adjustable elastic belt.^76,78^

The activPAL3^μTM^ posture sensor (9g; 2.35cm × 4.3cm × 0.5cm) uses proprietary algorithms to detect the inclination of the thigh, categorising behaviour into daily time spent sitting/lying (sedentary), standing and stepping, as well as the number of steps and sit-stand transitions. In this study, the activPAL3^μTM^ will be initialised using the manufacturer’s software, PAL Connect. The activPAL3^μTM^ will be attached with a waterproof, adhesive Tegaderm dressing to the right thigh of each participant, in a mid-anterior position.^37^

The positioning of both devices will be checked throughout the laboratory-based validation procedure (Objective 1). For Objectives 2 and 3, the researcher will instruct participants to remove the ActiGraph GT3X+ only during water-based activities (eg, bathing), and wear the activPAL3^μTM^ continuously. Participants will be asked to record dates and times of any device removal and replacement in logbooks.

### Data reduction and statistical analysis

#### Objective 1: Laboratory-based validation

*ActiGraph GT3X+ (criterion standard = indirect calorimetry).* Time-stamped raw data from the ActiGraph GT3X+ will be downloaded and exported into Microsoft Excel using Actilife, which will display the activity counts for each axis (Y, X, Z) and the VM, recorded per 1-second epoch. Each participant’s VO_2_ (ml·min·kg) data from indirect calorimetry will be graphed for each of the 11 activities, and the time period at which steady state VO_2_ is reached will be identified, allowing for variation ± .50 ml·min·kg (a total margin of 1.0 ml·min·kg). Once steady state VO_2_ periods have been identified, (eg, minutes 4–6), ActiGraph GT3X+ activity count data (Y-axis and VM) and METs (from indirect calorimetry), recorded during these steady state periods, will be extracted for statistical analysis.^79–81^ Where participants do not reach steady state VO_2_ during a specific activity, their data recorded during that activity will be excluded from statistical analysis.

ActiGraph GT3X+ activity counts (Y-axis and VM) and METs from each participant, per activity, will be averaged across the identified steady state VO_2_ time period for use in Receiver Operating Characteristic (ROC) curve analysis. This statistical test will be used to define both uniaxial (based on Y-axis activity counts) and triaxial (based on VM activity counts) accelerometer cut-points for sedentary time, and light and moderate intensity physical activity. The independent variable will be the average ActiGraph GT3X+ activity counts recorded during steady state VO_2_. Binary indicators (0 or 1) will classify the intensity of activities (as sedentary or moderate intensity physical activity), on the basis of average MET values recorded during steady state VO_2_ (dependent variable [*Table 2*]).

**Table 2.** The binary indicators that will be created in ROC curve analysis, using energy expenditure (METs) to classify the intensity of each activity for each participant.

Energy expenditure (METs)	Intensity of activity	Binary indicator
≤1.5	Sedentary	1
>1.5	>Sedentary	0
≥3	Moderate intensity physical activity	1
<3	<Moderate intensity physical activity	0

MET, metabolic equivalent of task

All MET values are based on the Sedentary Behaviour Research Network,^10,11^ and American College of Sports Medicine and the American Heart Association^82^

ROC curve analysis will be conducted using SPSS (IBM Corporation, Armonk, NY [version 24]). Each point on the ROC curve generated, will correspond to an activity count. Then, the activity count that maximises sensitivity (*y-*axis) and specificity (*x-*axis) will be identified using this curve. ROC curves will be generated for sedentary time and moderate intensity physical activity, on the Y-axis and VM. The activity counts representative of sedentary time and moderate intensity physical activity will correspond to the lower and upper threshold values for light intensity physical activity, respectively. Furthermore, the value corresponding to the area under the curve (AUC) will represent the accuracy, or ‘fit’, of the analysis, where-by 0.90–1.00 = excellent, 0.80–0.89 = good, 0.70–0.79 = fair, 0.60–0.69 = poor, and <0.60 = failure.

*ActivPAL3*^μ^*^TM^ (criterion standard = direct observation).* Time-stamped raw data recorded by the activPAL3^μTM^ during the laboratory protocol, will be downloaded and exported to Microsoft Excel using PAL Connect. Epoch data will be generated, to show time spent sedentary, standing or stepping every 15 seconds during the laboratory testing procedure, and the total number of steps and sit-stand transitions occurring during this period.

The researcher will observe the video camera recordings of each participant, and record behaviour during 15-second time intervals which correspond to the activPAL3^μTM^ 15-second epoch data generated by PAL Connect. Specifically, the researcher will record whether the participant was sitting/lying (sedentary), standing or stepping at every 15-second epoch, during each activity (standardised testing components and ADLs). These data will then be summed to determine total directly observed time spent sedentary, standing and stepping (minutes). The total number of steps and sit-stand transitions occurring throughout each activity (standardised testing components and ADLs) will also be recorded. Observed behaviours will be defined as: *sitting* – the participant’s back in an upright position, supporting their bodyweight through their buttocks; *lying* – the participant being horizontal on a surface; *standing* – the participant is upright with their feet supporting their body weight; *step (singular)* – the participant is in an upright position, and their foot has left the ground before making complete contact with the ground; *stepping* – continuous movement whilst in an upright posture.^11,64,68^

Using SPSS, means and standard deviations will be generated from activPAL3^μTM^ and direct observation data, to enable comparison between activPAL3^μTM^-assessed and directly observed time spent: 1) sedentary; 2) standing; 3) stepping (minutes), as well as the total number of steps and sit-stand transitions during the testing period. Bland-Altman plots will then be generated using SPSS, to evaluate the agreement between activPAL3^μTM^ estimates and direct observation of behaviours.

Finally, misclassification by the activPAL3^μTM^ of time spent sedentary, standing and stepping, as well as the number of steps and sit-stand transitions, will be calculated and reported as the percentage difference between activPAL3^μTM^-assessment and direct observation of behaviours.

#### Objective 2: Field-based validation of RA-specific accelerometer cut-points

Raw ActiGraph GT3X+ data will be downloaded and exported into Microsoft Excel using Actilife, which will display the activity counts for each axis (Y, X, Z) and the VM, recorded per 1-second epoch. To identify periods of non-wear, ≥60 and ≥90 minutes of consecutive ‘0’ counts, with a spike tolerance = 2 minutes, will be applied to the ActiGraph GT3X+ data. Data will be considered as valid for inclusion in subsequent statistical analysis, where participants have worn the accelerometer for ≥10 hours each day, for ≥4 weekdays, including ≥1 weekend day. The RA-specific sedentary time accelerometer cut-points derived during the laboratory-based validation (Objective 1) will then be applied to the free-living (7-day) ActiGraph GT3X+ data, to estimate time spent in sedentary behaviour (minutes/day [mean ± standard deviation]).

PAL Connect will be used to download and export activPAL3^μTM^ data, in 15-second epochs, to Microsoft Excel. Sleep time will be removed manually using information from wear-time logbooks, self-reported waking and sleeping time, and non-wear periods identified by Actilife (computed according to the aforementioned non-wear criteria). ActivPAL3^μTM^-assessed sedentary time will then be calculated (minutes/day [mean ± standard deviation]). Bland-Altman plots will be used to determine agreement between estimates of sedentary time assessed by the ActiGraph GT3X+ and activPAL3^μTM^, and bias and 95% limits of agreement will be calculated.

#### Objective 3: Accelerometer cut-point comparison

For Objective 3, estimates of sedentary time, and light and moderate intensity physical activity (minutes/day [mean ± standard deviation]), will be generated using the novel RA-specific accelerometer cut-points (Objective 1) and existing widely-used non-RA (uniaxial) accelerometer cut-points (Y-axis: sedentary time, ≤99 cpm; light intensity physical activity, 100–2019 cpm; moderate intensity physical activity, 2020–5998 cpm)^46,47^ Then, using the criterion of the activPAL3^μTM^, the validity of applying the non-RA accelerometer cut-point for measuring free-living sedentary time in RA, will be evaluated using Bland-Altman plots.

Using *t*-test analysis, estimates of sedentary time, and light and moderate intensity physical activity (minutes/day [mean ± standard deviation]), computed using RA-specific vs. non-RA accelerometer cut-points, will be compared within this sample of RA participants.

## DISCUSSION

The accurate assessment of sedentary time and physical activity among people living with RA is critical in order to understand the dose-response relationships between sedentary time and physical activity with RA outcomes. Numerous studies in non-RA populations have validated accelerometers against indirect calorimetry, developing population-specific accelerometer cut-points (eg, for children, adults and older adults), to provide a more valid means of quantifying sedentary behaviour and physical activity.^45,49,76^ More recently, the activPAL^TM^ has been reported to demonstrate high validity when compared against direct observation in several populations,^58,61,64,65^ and is considered the ‘gold standard’ measure of sedentary time.^37,58–63^ The current study will take the first steps to establish analytical procedures, that ensure widely-used objective devices can be employed to accurately measure sedentary time and physical activity in RA.

### Future research directions

Findings from this comprehensive validation study will therefore serve to direct future research employing activity count-based accelerometers (eg, ActiGraph) and the activPAL^TM^, to measure sedentary time and physical activity in RA. Specifically, this study’s results will provide guidelines for researchers when analysing these data. As such, results from this study will provide great potential for future research to more conclusively determine important relationships between sedentary time and physical activity, with pertinent RA outcomes and modifiable determinants of these behaviours, as well as evaluating the efficacy of interventions targeting sedentariness and physical activity in this patient group.

## ABBREVIATIONS

ADLs: Activities of daily living
AUC: Area under the curve
BMI: Body-mass index
cpm: Counts per minute
DAS-28: Disease Activity Score-28
METs: Metabolic equivalents
NHANES: National Health and Nutrition Examination Survey
RA: Rheumatoid Arthritis
ROC: Receiver Operating Characteristic
VM: Vector magnitude
